# Factors associated with initiation of antihyperglycaemic medication in UK patients with newly diagnosed type 2 diabetes

**DOI:** 10.1186/1472-6823-12-1

**Published:** 2012-03-07

**Authors:** Alan J Sinclair, Charles M Alexander, Michael J Davies, Changgeng Zhao, Panagiotis Mavros

**Affiliations:** 1 Beds & Herts Postgraduate Medical School, Luton, UK; 2 Merck Sharp & Dohme Corp, Whitehouse Station, NJ, USA; 3 Global Health Outcomes, Merck Sharp & Dohme Corp, Mail: WS2E76, 1 Merck Drive, Whitehouse Station, NJ 08889, USA

**Keywords:** Clinical inertia, Age, Type 2 diabetes mellitus, Antihyperglycaemic medication

## Abstract

**Aim:**

To assess the factors associated with antihyperglycaemic medication initiation in UK patients with newly diagnosed type 2 diabetes.

**Methods:**

In a retrospective cohort study, patients with newly diagnosed type 2 diabetes were identified during the index period of 2003-2005. Eligible patients were ≥ 30 years old at the date of the first observed diabetes diagnosis (referred to as index date) and had at least 2 years of follow-up medical history (N = 9,158). Initiation of antihyperglycaemic medication (i.e., treatment) was assessed in the 2-year period following the index date. Adjusted Cox regression models were used to examine the association between time to medication initiation and patient age and other factors.

**Results:**

Mean (SD) HbA_1c _at diagnosis was 8.1% (2.3). Overall, 51% of patients initiated antihyperglycaemic medication within 2 years (65%, 55%, 46% and 40% for patients in the 30- < 45, 45- < 65, 65- < 75, 75+ age groups, respectively). Among the treated patients, median (25^th^, 75^th ^percentile) time to treatment initiation was 63 (8, 257) days. Of the patients with HbA_1c _≥ 7.5% at diagnosis, 87% initiated treatment within 2 years. These patients with a higher HbA_1c _also had shorter time to treatment initiation (adjusted hazard ratio (HR) = 2.44 [95% confidence interval (CI): 1.61, 3.70]; p < 0.0001). Increasing age (in years) was negatively associated with time to treatment initiation (HR = 0.98 [95% CI: 0.97, 0.99]; p < 0.001). Factors significantly associated with shorter time to treatment initiation included female gender and use of cardiovascular medications at baseline or initiated during follow up.

**Conclusions:**

In this UK cohort of patients with newly diagnosed type 2 diabetes, only 51% had antihyperglycaemic medication initiated over a 2-year period following diagnosis. Older patients were significantly less likely to have been prescribed antihyperglycaemic medications. Elevated HbA_1c _was the strongest factor associated with initiating antihyperglycaemic medication in these patients.

## Background

Management of type 2 diabetes is centered on glycaemic control, in conjunction with controlling blood pressure, cholesterol, and other cardiovascular risk factors [1,2]. Diabetes treatment guidelines recommend initiating treatment with antihyperglycaemic medication either concomitantly with or following a brief period of lifestyle intervention [1,3]. Despite these recommendations and confirmed inadequate glycaemic control with lifestyle interventions, many patients with newly diagnosed type 2 diabetes remain untreated with antihyperglycaemic medication in clinical practice for extended periods of time [4-7]. This hyperglycaemic burden may have long-term consequences and increase the risk for both micro- and macrovascular complications of diabetes [8].

Early initiation of antihyperglycaemic medication is associated with reductions in microvascular events and long-term, legacy effects with reductions in myocardial infarction and death in patients with newly diagnosed type 2 diabetes [9-11]. Furthermore, treatment with antihyperglycaemic agents, as monotherapy, led to a 2- to 3-fold increase in the proportion of patients with an HbA_1c _< 7% relative to diet alone in patients with newly diagnosed type 2 diabetes [12]. Initiation of antihyperglycaemic medication and treatment targets should be based on clinical judgment following assessment of patient factors such as age, functional status, and pre-existing, co-morbid conditions and the risk of diabetes treatment-related side effects (e.g., hypoglycaemia) [1,3,8]. Given the increasing prevalence of type 2 diabetes in the UK [13], and the clinical benefits associated with antihyperglycaemic treatments, the present study was conducted to assess the association between patient age and initiation of antihyperglycaemic medication in UK patients following diagnosis of type 2 diabetes.

## Methods

### Data Source

The study cohort was drawn from the commercially-available, widely-used Intercontinental Medical Statistics (IMS) MediPlus database [14]. This database is composed of patient information from a representative sample of general practitioners and contains records of medical encounters, prescriptions, and enrollment data for approximately 3.7 million patients in the UK and Northern Ireland.

### Study Design and Patient Selection

In this retrospective analysis, patients ≥ 30 years old with newly diagnosed type 2 diabetes were identified using International Classification of Disease (ICD)-10 codes (E11-E14) during the index period of 2003 to 2005. Newly diagnosed status was defined as having no prior diagnosis of type 2 diabetes and no prescription for antihyperglycaemic agents in the database for at least 12 months prior to the first observed diagnosis (i.e., index date). The age cut-off was chosen to minimise the likelihood of selecting a patient with type 1 diabetes. Patients included in this study were continuously active in the database for at least 1 year preceding and 2 years following the index date.

### Analyses

The primary outcome was the proportion of patients who initiated antihyperglycaemic medication (also described as treatment or therapy for this analysis) during the 2-year period following the index date. Time to treatment initiation was calculated as the time between the index date and first prescription for any antihyperglycaemic agent during the follow-up period. Data were censored at 2 years of follow-up. The 2-year follow-up period was selected to allow for sufficient time to initiate medication following guideline treatment recommendations, while also maximizing the size of the cohort, since longer follow-up periods resulted in progressively smaller cohorts. Analyses were performed on the entire cohort with age as a continuous variable and by age groups (30- < 45, 45- < 65, 65- < 75, and 75+ years).

Patient baseline characteristics were assessed using data from the 12 months preceding the index date. Characteristics included age at the index date, gender, and co-morbid disease conditions. Limited laboratory data are available in the MediPlus database. For the present analysis, HbA_1c _values in the 6-month window centered on the index date (used as HbA_1c _at diagnosis [i.e., baseline] for analysis) were identified. This window was selected to maximise the number a patients with HbA_1c _values near the index date. Further, the potential 3-month period beyond the index date was chosen because the full effect of any diet or exercise intervention was not likely attained within this short time period. HbA_1c _values were also identified during the 6-month period prior to the end of the follow up (i.e., end of study period) for all patients that were not treated with antihyperglycaemic medications at the end of the study period. The objective was to assess the association between HbA_1c _values at the end of the study period and non-treatment.

Descriptive statistics (mean and standard deviations [SD] and proportions) were used for patient demographics, co-morbid conditions, and treatment characteristics. The Kaplan-Meier estimator of the survival function was used to demonstrate differences in the time to antihyperglycaemic treatment initiation across different age groups. Cox regression models were used to test for association between patient-related factors and time to initiation of antihyperglycaemic treatment adjusting for other predictors. Time-varying regression variables were included in the models to account for newly diagnosed conditions and newly prescribed (non-antihyperglycaemic) medications during the follow-up period. In the adjusted models, age was treated as a continuous variable and HbA_1c _at diagnosis was treated as a categorical variable (< 7.5%, ≥ 7.5%, or missing). The HbA_1c _cut point of 7.5% was selected because it is the high point of the range recommended in the UK for initiating antihyperglycaemic medication [15]. The chi-square test was used to test for significance.

## Results

In the MediPlus database, 43,486 patients had a diagnosis of type 2 diabetes. Of the 11,543 who had their first observed diagnosis between 2003 and 2005, 9,158 patients (54% male) met the inclusion criteria for this analysis. Mean (SD) age was 62.4 (12.8) years, with 9.6%, 44.3%, 27.5%, and 18.6% of patients within the 30- < 45, 45- < 65, 65- < 75, 75+ age groups, respectively (Table 1). HbA_1c _values were available for 55% (n/N = 5,044/9,158) of the entire cohort. There was no association between age and missing HbA_1c _values (p = 0.3876 for trend using chi-square test). Mean (SD) HbA_1c _at diagnosis was 8.1% (2.3) for the cohort of patients with HbA_1c _values, and 8.7% (2.4), 8.3% (2.3), 8.0% (2.2), and 7.7% (2.0) for those in the 30- < 45, 45- < 65, 65- < 75, 75+ age groups, respectively. The presence of pre-existing co-morbid conditions at baseline increased with age, except for liver disease where the opposite was observed (Table 1). During the follow-up period, newly diagnosed co-morbid conditions included cardiovascular conditions (5.7%), microvascular complications of diabetes (7.2%), cancer (1.8%), edema (1.7%), liver disease (0.5%), and Alzheimer's disease/dementia (0.2%). Use of antihypertensive and gastroprotective agents increased with age, whereas use of lipid-modifying agents was similar across age groups (Table 1). Newly prescribed medications during the follow-up included antihypertensive (10.1%), lipid-modifying (28.6%), weight-reducing (1.6%), and gastroprotective agents (6.3%).

**Table 1:** Baseline characteristics of patients with newly diagnosed type 2 diabetes during the index period

Variable	Entire Cohort N = 9,158		Age Group (years)		
		
		30 to < 45 n = 880	45 to < 65 n = 4,055	65 to < 75 n = 2,522	≥ 75 n = 1,701
Age, years (mean ± SD)	62.4 ± 12.8	38.9 ± 4.0	55.8 ± 5.4	69.3 ± 2.8	80.1 ± 4.2

Gender, male (%)	54	54	60	55	41

HbA_1c _at diagnosis, % (mean ± SD)	8.1 ± 2.3	8.7 ± 2.4	8.3 ± 2.3	8.0 ± 2.2	7.7 ± 2.0

Patients with HbA_1c _measurement at diagnosis, (n, (%))	5,044 (55.1)	481 (54.7)	2,274 (56.1)	1,365 (54.1)	924 (54.3)

**Patients with selected pre-existing conditions**					

Cardiovascular conditions (%)	19.2	2.5	13.4	25.4	32.4

Microvascular conditions (%)	1.7	0.5	1.0	1.8	3.8

Cancer (%)	4.2	1.3	2.1	5.6	8.4

Oedema (%)	2.5	0.6	2.3	2.7	3.6

Liver Disease (%)	0.6	1.0	0.6	0.5	0.2

Alzheimer/dementia (%)	0.3	0.0	0.1	0.3	1.0

**Patients with selected medication use**					

Antihypertensive agents (%)	59.1	21.4	52.6	69.3	78.4

Lipid-modifying agents (%)	27.8	34.3	24.2	36.4	34.3

Weight-reducing agents (%)	1.2	3.2	1.7	0.5	0.1

Gastroprotective agents (%)	23.3	15.2	20.0	27.2	29.5

**Figure 1:**
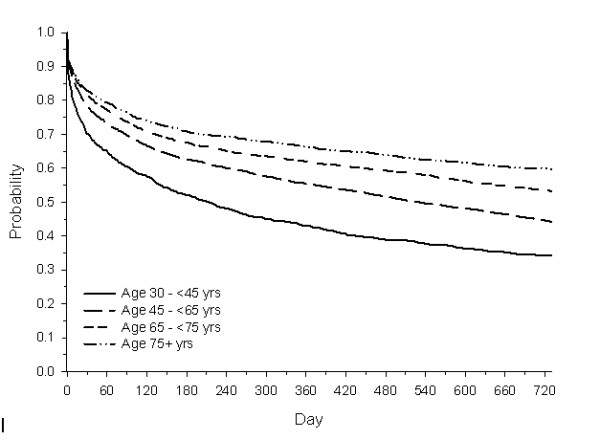
**Kaplan-Meier curves for time to initiation of antihyperglycaemic therapy after diagnosis of type 2 diabetes by age group**.

Overall, 36%, 42%, and 51% of patients initiated antihyperglycaemic therapy within 180 days, 1 year, and 2 years of diagnosis, respectively. The proportion of patients who had treatment initiated within 2 years of diagnosis decreased with advancing age (65%, 55%, 46%, and 40% for patients in the 30- < 45, 45- < 65, 65- < 75, 75+ age groups, respectively). The median time to treatment initiation increased with advancing age (213, 530, > 730, and > 730 days for patients in the 30- < 45, 45- < 65, 65- < 75, 75+ age groups, respectively [Figure 1]). Among the treated patients, median (25^th^, 75^th ^percentile) time to treatment initiation was 63 (8, 257) days, with treatment initiation increasingly delayed with age. Of the treatments prescribed, 76% of patients were prescribed metformin, 19% sulphonylurea, 4% insulin, and 1% other. Metformin use decreased with age (77%, 82%, 76%, and 66%, for patients in the 30- < 45, 45- < 65, 65- < 75, 75+ age groups, respectively; p < 0.0001 for trend using chi-square test) and sulphonylurea use increased with age (15%, 15%, 22%, and 32%, respectively; p < 0.0001 for trend using chi-square test).

Cox regression analysis adjusting for patients' baseline characteristics showed that increasing age was associated with longer time to initiation of antihyperglycaemic medication (Table 2). An HbA_1c _≥ 7.5% at diagnosis was associated with shorter time to treatment initiation (Table 2). In this cohort of patients (i.e., HbA_1c _≥ 7.5% at diagnosis, n = 2,446), 73%, 81%, and 87% initiated antihyperglycaemic therapy within 180 days, 1 year, and 2 years of diagnosis, respectively. There was a significant interaction between age and HbA_1c _at diagnosis such that the negative effect of age on treatment initiation was reduced in individuals with higher HbA_1c _values at diagnosis, i.e., ≥ 7.5% (Table 2). Other significant predictors associated with shorter time to antihyperglycaemic medication initiation included female gender, use of lipid-modifying agents, use of weight-reducing agents and later physician registration year. The missing indicator for HbA_1c _values was associated with shorter time to initiation (Table 2). During the follow-up period, development of cardiovascular conditions (Table 2), hospitalization, and new use of antihypertensive, lipid-modifying, gastroprotective, or weight-reducing agents were associated with shorter times to treatment initiation (Table 2).

**Table 2:** Adjusted hazard ratios for initiation of antihyperglycaemic treatment

Variable	Patient Sample (N = 9,158)	
	
	Hazard Ratio (95% CI)	P-value
*Baseline^a^*		

Age at first diagnosis, years	0.98 (0.97, 0.99)	< 0.0001

HbA_1c _≥ 7.5%	2.44 (1.61, 3.70)	< 0.0001

Dummy HbA_1c _(missing = 1)	1.62 (1.06, 2.47)	0.0247

Interaction: Age with HbA_1c _≥ 7.5%	1.015 (1.008, 1.022)	< 0.0001

Gender (male = 1; female = 0)	0.91 (0.86, 0.97)	0.0018

Physician registration years	1.007 (1.003, 1.011)	0.0005

Lipid-modifying agents	1.22 (1.12, 1.32)	< 0.0001

Weight-reducing agents	1.59 (1.27, 1.99)	< 0.0001

*New at follow up*		

Cardiovascular conditions	1.36 (1.16, 1.59)	0.0001

Antihypertensive agents	1.43 (1.28, 1.61)	< 0.0001

Lipid-modifying agents	2.41 (2.21, 2.62)	< 0.0001

Gastroprotective agents	1.59 (1.36, 1.88)	< 0.0001

Weight-reducing agents	1.38 (1.06, 1.81)	0.0175

Hospitalization	1.37 (1.22, 1.54)	< 0.0001

*^a^*Baseline variables assessed in 12-month period prior to diagnosis of type 2 diabetes. Non-significant predictors included in the model were indicators for baseline cardiovascular conditions, microvascular conditions, cerebrovascular conditions, cancer, liver disease, Alzheimer's disease/dementia, peptic ulcer disease, rheumatic conditions, chronic pulmonary disease, hemi- or paraplegia, oedema, antihypertensive medication use, and gastroprotective medication use, and indicators for follow-up microvascular conditions, cancer, oedema, liver disease and Alzheimer's disease/dementia.

**Figure 2:**
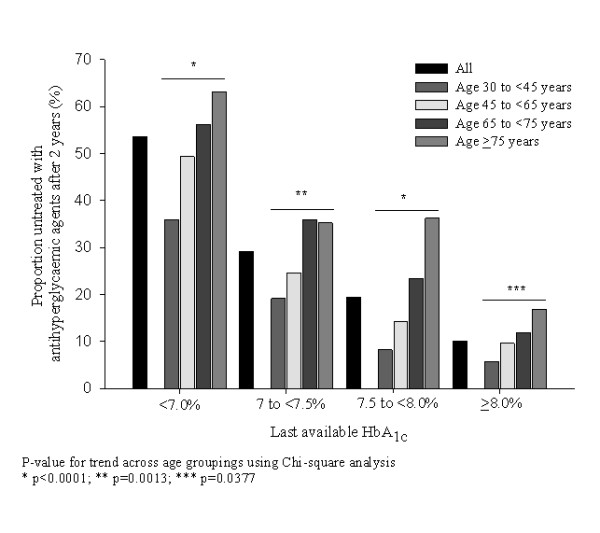
**Proportion of patients untreated with antihyperglycaemic medication after the 2-year follow-up period by age group and last available HbA_1c _value**.

Figure 2 illustrates that higher HbA_1c _values at the end of follow up were associated with lower levels of non-treatment with antihyperglycaemic medications. Within each HbA_1c _category there was a significant trend for patients in the older age groups to remain untreated (Figure 2). Among those untreated, the proportion of patients with an HbA_1c _≥ 7.5% was not statistically different across age groups (p > 0.05).

## Discussion

The present study assessed the time to antihyperglycaemic medication initiation in a UK cohort of patients with newly diagnosed type 2 diabetes and found that the proportion of patients who had antihyperglycaemic therapy initiated after 2 years of follow up was 51%, with lower rates of treatment initiation observed in older compared to younger individuals. This percentage is less than the 75% of Dutch patients with type 2 diabetes who initiated oral antihyperglycaemic therapy within 2 years of diagnosis [6]. In a Danish cohort, 70% of newly diagnosed diabetic patients received antihyperglycaemic therapy after nearly 6 years of follow up, despite a mean baseline HbA_1c _level of 10.2% [5]. Glycaemic control has been shown to deteriorate over time in patients with newly diagnosed type 2 diabetes, specifically those untreated with antihyperglycaemic medication (i.e., receiving only diet and lifestyle intervention) [12,16]. Therefore, given that the mean HbA_1c _was ~8.0% around the time of diagnosis for those with measurements and in the absence of other factors, more patients should have been initiated on antihyperglycaemic medications over the 2-year period than the 51% observed in the present study.

Although various algorithms for treatment of type 2 diabetes were in place or introduced during the time period assessed for our study (2003 - 2007), the recommendations are generally similar for patients with newly diagnosed type 2 diabetes [15,17]. The recommendations include language stating that lifestyle modifications should be initiated with follow-up assessment of glycaemic control (i.e., fasting glucose and HbA_1c_) within a 3 to 6 month period. If HbA_1c _targets are not achieved with lifestyle modifications, initiation of antihyperglycaemic medication should be considered along with continuation of lifestyle changes. Despite such recommendations, this study demonstrated that 2 years after initial diagnosis of type 2 diabetes, a large proportion of patients remain untreated. The proportion of untreated patients was inversely related to the HbA_1c _values with a greater proportion of patients receiving treatment as HbA_1c _increased. However, 30% of patients with an HbA_1c _value ≥ 7.5% at the end of follow up had not yet received treatment, despite the apparent need for treatment based on guidelines.

Management of type 2 diabetes is related to a myriad of patient-, physician, and systematic-related factors [18]. Patient age may affect treatment initiation or intensification and limit treatment choices because of the increased likelihood of co-morbidities and frailty in older patients [19]. In the present study older patients were more likely to have pre-existing, co-morbid conditions. After adjusting for these differences, increasing age was still associated with a decreased likelihood of physician prescribing of antihyperglycaemic medication. Similar findings were found with a US cohort of patient with newly diagnosed type 2 diabetes [20]. Conversely, higher HbA_1c _values near the time of diagnosis increased the likelihood of a physician initiating antihyperglycaemic medication. Younger patients had higher HbA_1c _at diagnosis, which account for part of the higher rates of treatment initiation relative to older patients. When controlling for HbA_1c _values at diagnosis, older patients were less likely to initiate treatment than younger patients. However, a significant interaction was observed between age and HbA_1c _values ≥ 7.5% at diagnosis, suggesting that the influence of age on non-treatment with antihyperglycaemic medication was reduced as HbA_1c _increased above 7.5%. Similar trends were observed when HbA_1c _at the end of follow up was used in the analysis. It is apparent that older patients in this study were not treated as frequently with antihyperglycaemic therapy as younger patients with the same HbA_1c _level. A recent survey study evaluated the reasons UK general practitioners do not treat their newly diagnosed type 2 diabetes patients with antihyperglycaemic medications. Reasons cited by the general practitioners were those related to adequate glycaemic control for both younger and older patients. However, issues related to safety of antihyperglycaemic agents, burden to the patients, or cognitive or physical function of the patient were selected more often by GPs for not treating their older patients [21]. Collectively, the present findings are consistent with the less stringent, glycaemic target recommendations for older adults, especially those with pre-existing, co-morbid conditions [22].

In addition, the development or treatment of co-morbid conditions during the follow-up period was positively associated with initiating antihyperglycaemic treatment. The new conditions may have prompted the physician to evaluate glycaemic control in the context of increased risk factors for cardiovascular disease, as recommended by treatment guidelines [1,15]. Of the patients who initiated treatment, median time to start treatment was approximately 2 months. This is consistent with clinical guidelines in place at the time (2003 - 2007) that suggested initiating antihyperglycaemic treatment if inadequate glycaemic control was present after a short period (3 to 6 months) of lifestyle intervention [15,17].

These limitations should be considered when interpreting the present results. HbA_1c _measures around the time of diagnosis were available for only 55% of the patients. Thus, the HbA_1c _results may not reflect the true baseline value at the time of diagnosis for the entire cohort. The study had only a 2-year follow-up period. If one more year of follow up was added, the patient count would have been reduced by 15-30%. Although eligible patients had to be at least 30 years old and were identified using ICD-10 codes for type 2 diabetes, some patients with type 1 diabetes may have been incorrectly indentified as having type 2 diabetes, although the number is likely to have been small.

In summary, in this UK cohort of patients with newly diagnosed type 2 diabetes, only 51% had antihyperglycaemic medication initiated over a 2-year period following diagnosis, with older patients significantly less likely to have been prescribed medication by their physicians. Elevated HbA_1c _was the strongest factor associated with initiating antihyperglycaemic medication in these patients. These results highlight the under-treatment of older adults with type 2 diabetes. Further research is needed to better understand the reasons for the observed differences between younger and older patients with type 2 diabetes.

## Competing interests

CMA, MJD, CZ, and PM are employees of Merck Sharp & Dohme, Corp. AJS has no conflict of interest related to this analysis.

## Authors' contributions

AJS, CMA, CZ, and PM were involved in the concept and design of the study. CZ and PM were involved in the data collection and/or analysis. All authors were involved in interpretation of the results. MJD and PM drafted the article and all authors were involved in the critical revision and approval of the article.

## Pre-publication history

The pre-publication history for this paper can be accessed here:

http://www.biomedcentral.com/1472-6823/12/1/prepub
